# Active targeting schemes for nano-drug delivery systems in osteosarcoma therapeutics

**DOI:** 10.1186/s12951-023-01826-1

**Published:** 2023-03-22

**Authors:** Pengzhi Shi, Zhangrong Cheng, Kangcheng Zhao, Yuhang Chen, Anran Zhang, Weikang Gan, Yukun Zhang

**Affiliations:** Department of Orthopedics, Union Hospital, Tongji Medical College, Huazhong University of Science and Technology, Wuhan, 430022 China

**Keywords:** Osteosarcoma, Nano-drug delivery system, Active targeting, Passive targeting, Targeting ligand

## Abstract

Osteosarcoma, the most common malignant tumor of the bone, seriously influences people’s lives and increases their economic burden. Conventional chemotherapy drugs achieve limited therapeutic effects owing to poor targeting and severe systemic toxicity. Nanocarrier-based drug delivery systems can significantly enhance the utilization efficiency of chemotherapeutic drugs through targeting ligand modifications and reduce the occurrence of systemic adverse effects. A variety of ligand-modified nano-drug delivery systems have been developed for different targeting schemes. Here we review the biological characteristics and the main challenges of current drug therapy of OS, and further elaborate on different targeting schemes and ligand selection for nano-drug delivery systems of osteosarcoma, which may provide new horizons for the development of advanced targeted drug delivery systems in the future.

## Introduction

Osteosarcoma (OS) is the most common orthotopic bone tumor, accounting for approximately 35% of all primary bone malignancies. The male-to-female ratio is nearly 1.5:1.0, and 80–90% of OS cases occur in the long tubular epiphysis of the extremities, with the proximal tibia of the distal femur and proximal humerus being the most common site. Routine therapies include early surgical resection and adjuvant chemotherapy treatments, which have increased the 5-year survival rate to 60–70%, but 40% of patients still respond poorly to treatment [1, 2]. Current first-line chemotherapeutic agents include doxorubicin (DOX), cisplatin (CDDP), and methotrexate (MTX), which synergistically exert tumor cell-killing effects through different mechanisms of action [3]. Owing to the absence of selectivity and sensitivity to tumor cells and the application of high doses, chemotherapy drugs often have systemic toxicity such as cardiomyopathy, emesis, and alopecia [4, 5]. Therefore, there is an urgent need to explore novel therapeutic options for OS.

In the last decade, the application of nanotechnology in medicine has demonstrated several advantages. Nanocarrier-based drug delivery platforms have been extensively studied and offer new perspectives for the treatment of OS. Various nano-drug delivery systems, such as nanoparticles (NPs), liposomes, micelles, dendrimers, and nanogels, have been designed to deliver chemotherapeutic drugs and address the limitations of conventional chemotherapy [6–10]. Nano-drug delivery systems have the following benefits: (1) increasing the solubility of some hydrophobic drugs, (2) protecting drugs from degradation and prolonging blood circulation, (3) modifying nanocarriers to enhance targeted drug delivery and controlled release and improving drug bioavailability, and (4) improving multidrug delivery or multi-protocol synergistic therapy, such as chemotherapy combined with photothermal therapy (PTT) [11, 12].

**Fig. 1:**
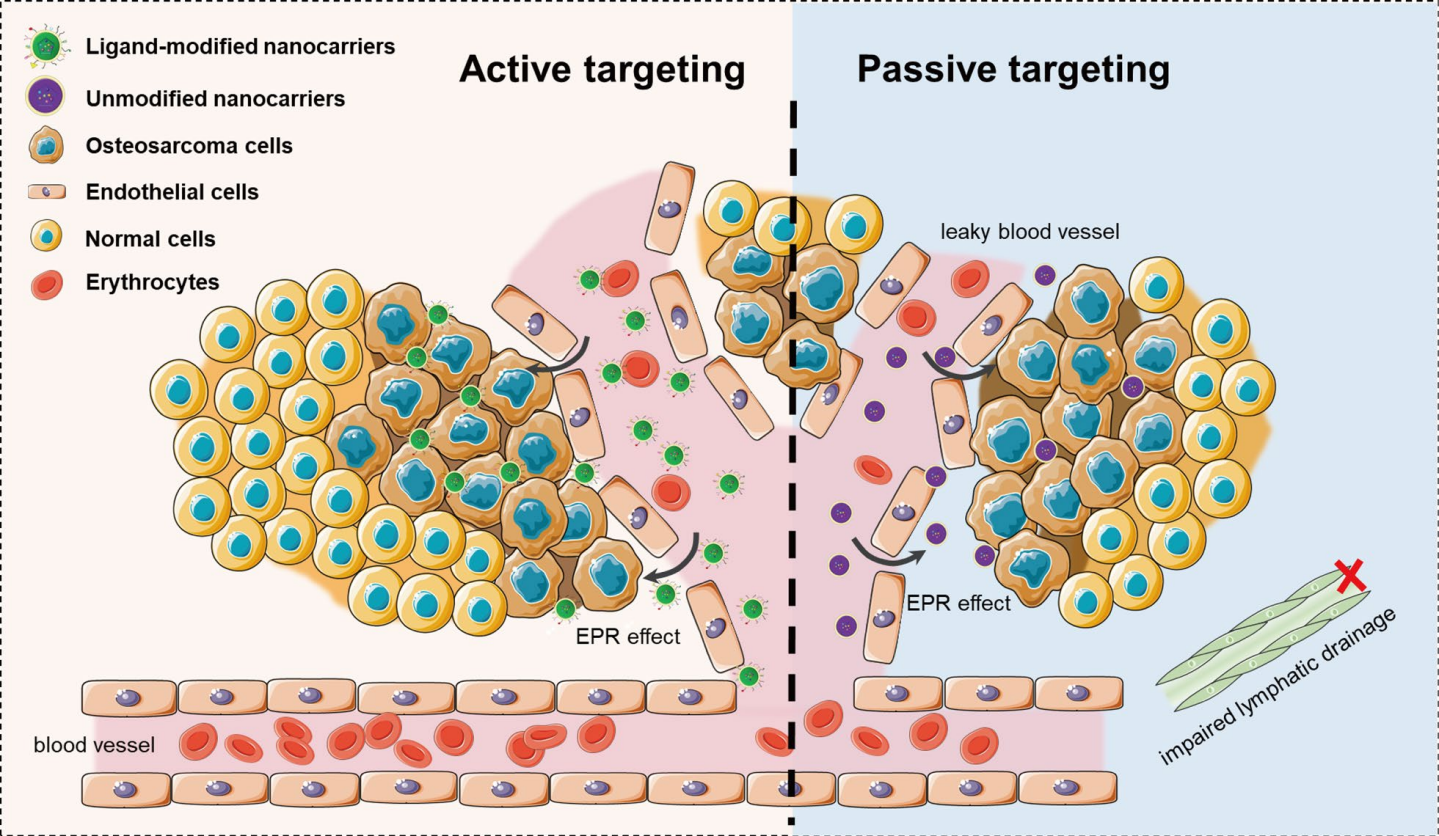
A schematic illustration of active targeting and passive targeting of nano-delivery system in anti-tumor therapy. Passive targeting is achieved via enhanced permeability and retention (EPR) effects. Nanocarriers circulate in the bloodstream, extravasate, and accumulate into tumor tissue through the leaky tumor vasculature. Targeting ligand-modified nanocarriers are able to bind to highly expressed receptors on the tumor cell, exerting local drug delivery or internalization through receptor-mediated endocytosis

Current accumulation schemes of nano-drug delivery systems are mainly divided into two types: passive targeting and active targeting (Fig. 1) [13]. Owing to vascular system leakage and impaired lymphatic clearance, small-molecule compounds undergo efficient extravasation and retain in the tumor interstitium (the enhanced permeability and retention (EPR) effect) [14]. Passive targeting refers to the passive accumulation of small molecules through the EPR effect to achieve therapeutic effects in tumor tissues. Whereas active targeting means conjugating targeting moieties, such as aptamers, ligands, and antibodies, on the surface of nanocarriers to specific recognition of tumor cells [15]. By specifically recognizing overexpression receptors on the surface of tumor cells, receptor-mediated endocytosis to internalize nanocarriers is stimulated, thereby generating higher therapeutic efficacy [16]. Various targets, such as folate receptors, vascular endothelial growth factor receptors (VEGFRs), and integrins, have been applied in nano-drug delivery systems for targeting OS [17–19]. In this paper, we first describe the biological characteristics and the main challenges of current drug therapy of OS, and further elaborate on active targeting schemes and ligand selection in nano-drug delivery systems.

## The biological characteristics of OS

As the most common primary malignant bone tumor, the incidence of OS is significantly higher in both age groups: children and adolescents aged 10–14 years, and older people aged 70–80 years [20, 21]. The main symptoms of OS patients including decreased range of motion, intense and painful swelling, and pathological fracture [22]. Approximately 10–20% of patients have metastases, predominantly in the lungs, at the time of their initial presentation [23]. This malignant bone tumor is histologically characterized by a mass of malignant pleomorphic osteoblasts and secretion of immature tumor osteoid matrix compared with other soft tissue sarcomas [24]. However, the immature osteoid matrix is not as strong as normal bone and therefore is not capable of handling the demands of daily activities. Moreover, the rapidly proliferating cells abnormally alter the kinetics of bone remodeling. On the one hand, osteoclast activity associated with abnormal RANK/RANKL signaling increases, leading to the development of osteolytic resorption, on the other hand, extracellular vesicles secreted by OS cells are able to promote osteolysis, leading to the development of aggressive osteolytic lesions, which leads to an increased risk of bone fragility and pathological fractures [25, 26]. In addition, OS erodes into the surrounding soft tissues through the osteocortex and endothelium, causing local compression and soft tissue swelling, which seriously affects people's normal life.

## Drug delivery challenges and barriers in OS

The specific tumor microenvironment of OS acts as a protective barrier severely hindering the effective delivery of drugs. Firstly, huge histological heterogeneity and genomic instability of OS determine the complexity and inefficiency of treatment. Whole genome sequencing revealed a highly rearranged genomes of OS with extensive copy-number aberrations and frequent disruption of tumor suppressors. Genetic chaos displayed on the macroscopic level in osteosarcoma include chromosome duplications, chromothripsis and kataegis [27]. Given this, targeted therapies based on genetic feature is challenging. Secondly, the dense bone matrix contains a large amount of inorganic minerals, which may restrain the penetration and accumulation of drugs in bone tumors [5, 28, 29]. And a low blood flow distribution (blood-bone marrow barrier) also results in drug limited delivery to the bone and ultimately leads to a compromised killing effect in OS [30]. Thirdly, multidrug resistance acquired by tumor is another important barrier that limits drug delivery. The most frequent mechanisms of multidrug resistance include increased expression of enzymes related to drugs metabolism, particularly glutathione S-transferases, overexpression of drug transporters and efflux proteins, and point mutations in proteins that are drug targets [31]. Lastly, deficiency of definite targeting biomarkers often induces fail to accumulate antitumor drugs at the sites of bone tumors. The non-specific biodistribution of chemotherapeutic drugs often induces adverse effects on the patients. Although nano-drug delivery systems passively target tumor tissues through the EPR effect, limitations such as randomness of targeting and low efficiency of drug diffusion to tumor cells make the implementation of passive targeting strategy unsatisfactory [32]. Active targeting tackles a portion of the systemic adverse effects due to non-specific distribution through ligand modification, enabling more efficient and specific accumulation in bone tumors.

## Active targeting

Nanocarriers modified with targeting ligands allow for precise spatial control in vivo, which significantly improves the efficacy of chemotherapeutic agents that are compromised by passive accumulation and the inability to specifically identify tumor cells [33]. Various biomarkers are specifically expressed or highly expressed on the surface of tumor cells, and the ligand-modified nanocarrier system can efficiently identify tumor cells by binding to these markers, which will greatly reduce damage to normal tissues. For the treatment of OS, active targeting involves the application of peripherally conjugated targeting ligands for enhanced delivery of nanocarriers. Depending on the target binding site, we divided the active targeting schemes into organ targeting (bone tissue targeting) and OS cells targeting, and provided a detailed description of the targets and ligands according to the functions and characteristics involved.

### Bone tissue targeting

**Fig. 2:**
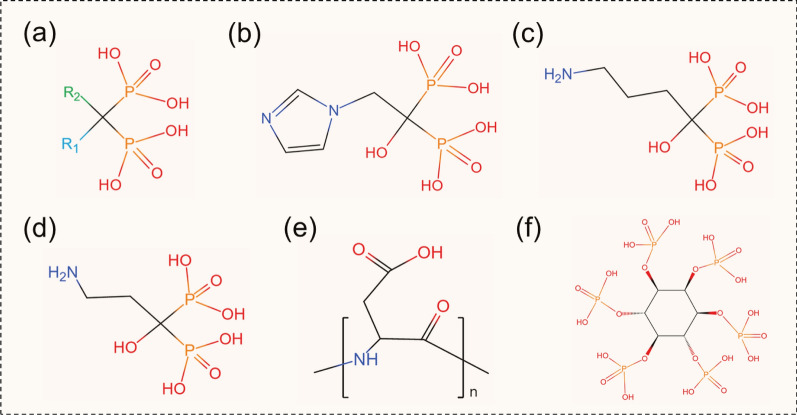
Molecular structures of bone tissue targeting ligands. **a** BPs; **b** ZOL; **c** ALN; **d** pamidronate; **e** aspartic acid-rich peptides; **f** phytic acid

Bone is composed of inorganic minerals (50–70%), organic matrix (20–40%), and water and fat (10%) [34]. The organic matrix is mainly collagen, which gives value to the elastic resistance of the bone and serves as a substrate for mineral deposition and growth [35]. Inorganic minerals are formed by the deposition of several types of calcium phosphate mineralization. As the most thermodynamically stable crystalline phase of calcium phosphate in body fluids, hydroxyapatite (Ca_10_(PO_4_)_6_(OH)_2_; HAp), the main component of inorganic minerals in bone tissue, becomes an ideal target for bone [36]. Small-molecule compounds have a high affinity for HAp, such as bisphosphonates (BPs), phytic acid (PA), aspartic acid-rich peptides, and tetracyclines, and these anion-rich ligands bind by chelating superficial calcium ions on the HAp surface (Fig. 2, Table 1).

**Table 1:** Examples of bone tissue targeting

Targeting ligand	Nanocarrier type	Agent	Cell lines	References
ALN	Liposomes	DOX	K7M2 OS cells 4T1murine breast cancer cells RAW264.7 murine monocyte-macrophage cells	[7]
	HPMA copolymer	TNP-470	K7M2 OS cells	[45]
	Nanoclusters	DOX	HOS/MNNG OS cells	[49]
	NPs	SPION	K7M2 OS cells	[50]
ZOL	NPs	Gemcitabine & Epirubicin	MG-63 cells	[53]
	NPs	DOX & DOXY	OS-732 cells	[54]
Pamidronate	Polylactide NPs	DOX	K7M3 OS cells and GFP-labeled K7M2 OS cells	[51]
Asp8	Micelles	DOX	Saos-2 OS cells	[63]
	NPs	Platinum-copper alloy	MDA-MB-231 cells	[65]
	Boronated polymer	Saporin	143B OS cells and MDA-MB-231 cells	[67]
PA	NPs	Cisplatin	MDA-MB-231 cells	[74]
	NPs	Platinum	NIH3T3 cells and PC-9 cells	[75]

*ALN* alendronate, *ZOL* zoledronate, *PA* phytic acid, *NPs* nanoparticles, *DOX* doxorubicin, *SPION* superparamagnetic iron oxide nanoparticle, *DOXY* doxycycline, *OS* osteosarcoma

#### Bisphosphonates

BPs are a class of compounds widely used in the treatment of diseases related to bone metabolism and have shown good efficacy in the treatment of hypercalcemia, osteoporosis, and Paget’s disease by inhibiting osteoclast differentiation and reducing bone resorption [37–39]. BPs functions depend largely on the backbone structure of the two terminal phosphate groups bound by the central carbon atom (P-C-P), which generated a high affinity through electrostatic interactions with Ca^2+^ of the HAp by bi- or tridentate chelation [40]. The affinity and pharmacological activity of BPs are also influenced by the covalent R1 and R2 side chains **(**Fig. 2a). For example, the hydroxyl group occupying the R1 side chain position can trigger tri-dentate chelation with HAp, while the nitrogen group modification at the R2 side chain can interact with the hydroxyl group on hyaluronic acid (HA) through hydrogen bonding [41, 42]. Three generations of BPs are distinguished based on whether the R2 side chain contains nitrogen. First-generation BPs, such as etidronate, chlorophosphonate, and tiludronate, are nitrogen-free; second-generation BPs, such as alendronate (ALN) and pamidronate, are nitrogen-containing; third-generation BPs, such as zoledronate (ZOL) and risedronate, are nitrogen-containing heterocycles [43].

Owing to the P-C-P structure, BPs exhibit targeting properties to bone tissue and have been widely applied in clinical practice. For example, radioisotope-labeled BPs (99mTc-HDP) have been developed for bone imaging [44]. Several BPs-modified multifunctional nano-drug delivery systems were recently developed for targeted OS therapy, among which ALN-modified NPs are the most commonly investigated. Segal et al. [45] first combined ALN with the potent antiangiogenic agent TNP-470 and N-(2-hydroxypropyl) methacrylamide copolymer to construct nanoscale conjugate. This conjugate showed antitumor efficacy by specifically inhibiting tumor-induced neovascularization and reducing the systemic toxicity of the free drug.

**Fig. 3:**
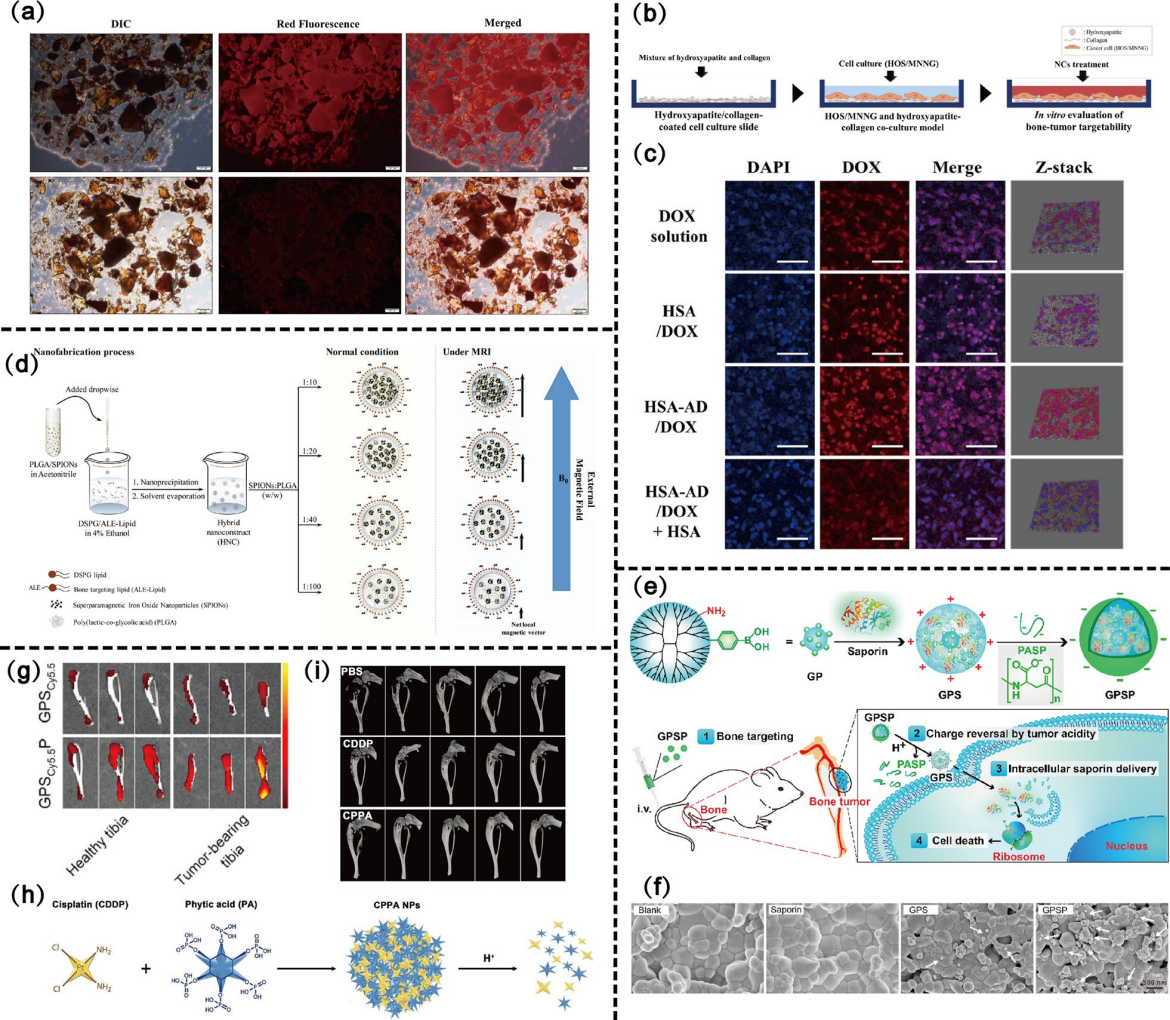
Bone tissue targeting scheme design and efficacy assessment of nano-drug delivery systems. **a** Representative fluorescence images of HAp crystal after incubation with RhB-labeled TNPs (upper panel) showing the interaction between HAp and targeted NPs. DIC: Imaging of HAp crystal. Copyright 2016, Scientific Reports. **b** Scheme of the HOS/MNNG and hydroxyapatite collagen co-culture model. **c** Confocal microscopy images of HOS/MNNG cells co-cultured with HAp and collagen matrix. Copyright 2022, Journal of Controlled Release. **d** Schematic represents the fabrication process of HNC using nanoprecipitation. Copyright 2017, Nanoscale. **e** Design of bone-targeted protein nanomedicine for the treatment of malignant bone tumors. **f** GPS or GPSP nanoparticles adsorbed on the HAp tablets. **g** Fluorescence images of tibias from healthy mice and bone tumor-bearing mice incubated with GPSCy5.5 and GPSCy5.5P, respectively. *GP* G5-PBA molecule, *GPS* GP/saporin complex. Copyright 2022, Bioactive Materials. **h** Schematic illustration of CPPA nanoparticles. **i** 3D micro-CT reconstruction of the tibias after treatment. Copyright 2020, Small

Anthracyclines, such as DOX, are first-line drugs for the treatment of OS. The mechanisms by which DOX causes cellular damage include intercalation into DNA and disruption of topoisomerase II-medicated DNA topology during replication and transcription, and cell membrane (CM) damage through generation of free radicals, leading to cancer cell death [46]. DOX-loaded liposomal NPs modified with ALN improved the internalization of 143B OS cells [47]. The strong interaction property of ALN with the bone was used to deliver NPs into the bone microenvironment. NPs accumulate significantly in HAp crystals after the use of a bone model of HAp crystals to assess the bone-targeting efficiency of nano-drugs in vitro [48] (Fig. 3a). Kang et al. [49] selected albumin as the core material and constructed an albumin nano-drug delivery system (Human serum albumin (HSA) was decorated with alendronate (AD), and DOX-loaded nanoclusters (HSA-AD /DOX)) to reduce the systemic adverse effects of DOX. This study simulated the bone tumor microenvironment using the human OS cell line *N-*methyl-*N'*-nitro-*N*-nitrosoguanidine–human OS (MNNG-HOS) and HAp gum co-culture model to evaluate the targeting ability of HSA-AD/DOX (Fig. 3b). The results confirmed that the affinity of HSA-AD/DOX for HAp-collagen matrix was fivefold higher than that of HSA/DOX, and suggested that the nanoclusters via ALN modification are a promising delivery system for bone tumor treatment (Fig. 3c). Superparamagnetic iron oxide NPs (SPIONs)-based clinical diagnostic contrast agents have a short circulating half-life, lack disease specificity, and have a low transverse magnetic relaxation range. ALN-modified SPIONs also demonstrated good bone-targeting effects. As shown in Fig. 3d, the bone cancer-targeted hybrid nanoconstruct (HNC) modified with ALN demonstrated enhanced bone affinity, improved transverse magnetic relaxation rate, and showed enhanced contrast with surrounding soft tissues in magnetic resonance imaging of bone tumor-bearing mice [50].

In addition to ALN, other BPs-coupled NPs such as pamidronate and ZOL have demonstrated good bone-targeting effects. Yin et al. [51] designed pamidronate-functionalized NPs to delivery DOX into the bone microenvironment. The combination of pamidronate-mediated active bone targeting and passive targeting mediated by the EPR effect of NPs significantly increased the accumulation of DOX-loaded NPs in OS [52]. Modified ZOL-loaded multidrug PH-sensitive nanocarriers achieved 89% absorbtion in bone powder in 6 h, and exerted a dual-drug synergistic effect with 88% cell viability inhibition compared with single or free drugs [53]. To further compare the bone-targeting efficiency of different BPs-modified NPs, Tong et al. evaluated NPs formed by four BPs compounds (etidronate disodium, pamidronate disodium, alendronate disodium, and zoledronic acid) covalently bound to mesoporous silica NPs (MSN). The results confirmed that ZOL was the most potent bone-targeting fraction among the four BP compounds [54]. Overall, BPs are tissue-specific as targeted ligands for osteosarcoma treatment. Bone tissue targeting overcomes the low penetration and accumulation efficiency of nano-drugs in bone tissue and offers the prospect for drug delivery in osteosarcoma.

#### Aspartic acid-rich peptides

Non-collagenous proteins, such as osteocalcin and osteopontin, have a high affinity for bone tissue because they are rich in aspartic acid (Asp). The mechanism of Asp oligopeptide bone affinity is the ionic interaction between the polycarboxyl group of the peptide providing a negative charge and calcium ions in the mineral component of the bone (HAp) [55]. Therefore, Asp-rich peptides are promising bone tissue targets [56]. Current Asp-based peptide-modified nano-drug delivery systems have demonstrated good bone-targeting potential in studies on fractures, osteoporosis, bone tumors and metastases, and bone infections [57–61]. The bone affinity of Asp-rich peptides depends on the number of exposed amino acid residues. The higher the number of exposed Asp residues, the stronger the bone affinity [62]. Among them, the d-Asp octapeptide (Asp8), composed of eight Asps, is the most commonly used sequence for bone-targeted drug delivery [63, 64]. PTX-Asp8-Lip showed the highest affinity because of the higher number of aspartate residues exposed on the liposome surface. The binding capacity of Asp8-modified NPs to HAp or bone tissue can be increased by several times [65, 66]. Asp8-modified nanomicelles (DOX-A1/2-D8) reached a 91% binding rate of HAp compared with 55% of DOX-A4-K-D4, which promoted DOX accumulation in OS and reduced the side effects of chemotherapy [63]. Dendritic platinum–copper alloy NPs (Asp-DPCN) modified by Asp8 exerted good PTT while effectively accumulating in the bone tissue around bone tumors [65].

GPSP NPs constituted by the co-assembly of saponins (a toxin protein) with boronized polymers and the bone-targeting polymer poly-(α,β)-DL-aspartate (PASP) are used for intracellular protein drug delivery, which maintains the biological activity and protects protein drugs from degradation [67] (Fig. 3e). Importantly, the charge characteristics of PASP-coated GPSP NPs changed from positive to negative, thus avoiding rapid clearance of cationic NPs by the reticuloendothelial system during circulation [68]. PASP showed good bone-targeting properties both on the HAp surface and tumor-bearing tibia (Fig. 3f, g).

Using Asp-rich peptide as a bone-seeking agent is a charming option because they have no significant adverse effects [69]. More studies should be conducted to investigate the bone-targeting effects of Asp-rich peptides.

#### Phytic acid

PA, also known as inositol hexaphosphate, is a naturally occurring polyphosphorylated carbohydrate found in many high-fiber foods, such as beans, vegetables, and fruits [70, 71]. Interestingly, the molecular structure of PA contains six phosphate groups, suggesting that it may have bone-targeting abilities similar to BPs [72]. Moreover, PA exhibits inherent anticancer ability [70, 73]. CDDP crosslinked PA (CPPA) NPs formed after the reaction of the phosphate group of PA with cis-Diaquodiammineplatinum (II) can produce the ability of bone targeting via the residual phosphate group chelating with calcium ions on bone tissues (Fig. 3h). CPPA NPs had a 2.5-fold higher ability to bind HAp sheets than CDDP [74]. Meanwhile, they showed higher drug loading and pH-responsive drug-release behaviors and demonstrated effective anticancer effects on animal models (Fig. 3i). Similarly, Zhou et al. [75] prepared PA-coated platinum NPs (PA/PtNPs), which showed a threefold increase in bone-binding affinity after PA modification, providing a basis for the subsequent combination of PA-related anticancer and PTT.

NPs modified with bone-targeting ligands such as tetracyclines and PA have been studied more in osteoporosis and osteoarthritis, whereas nano-drug delivery systems applied to OS are relatively few. Thus, this will be a direction for future OS-targeting research [76–78]. However, bone-targeted ligands target bone tissue, not OS, and long-term residence in bone tissue may affect osteoclast activity and bone homeostasis [56, 79]. Perhaps the construction of dual-targeted nano-drug delivery systems (bone-targeted and OS-targeted) would better overcome the current barriers to bone tissue delivery.

### OS cell targeting

**Fig. 4:**
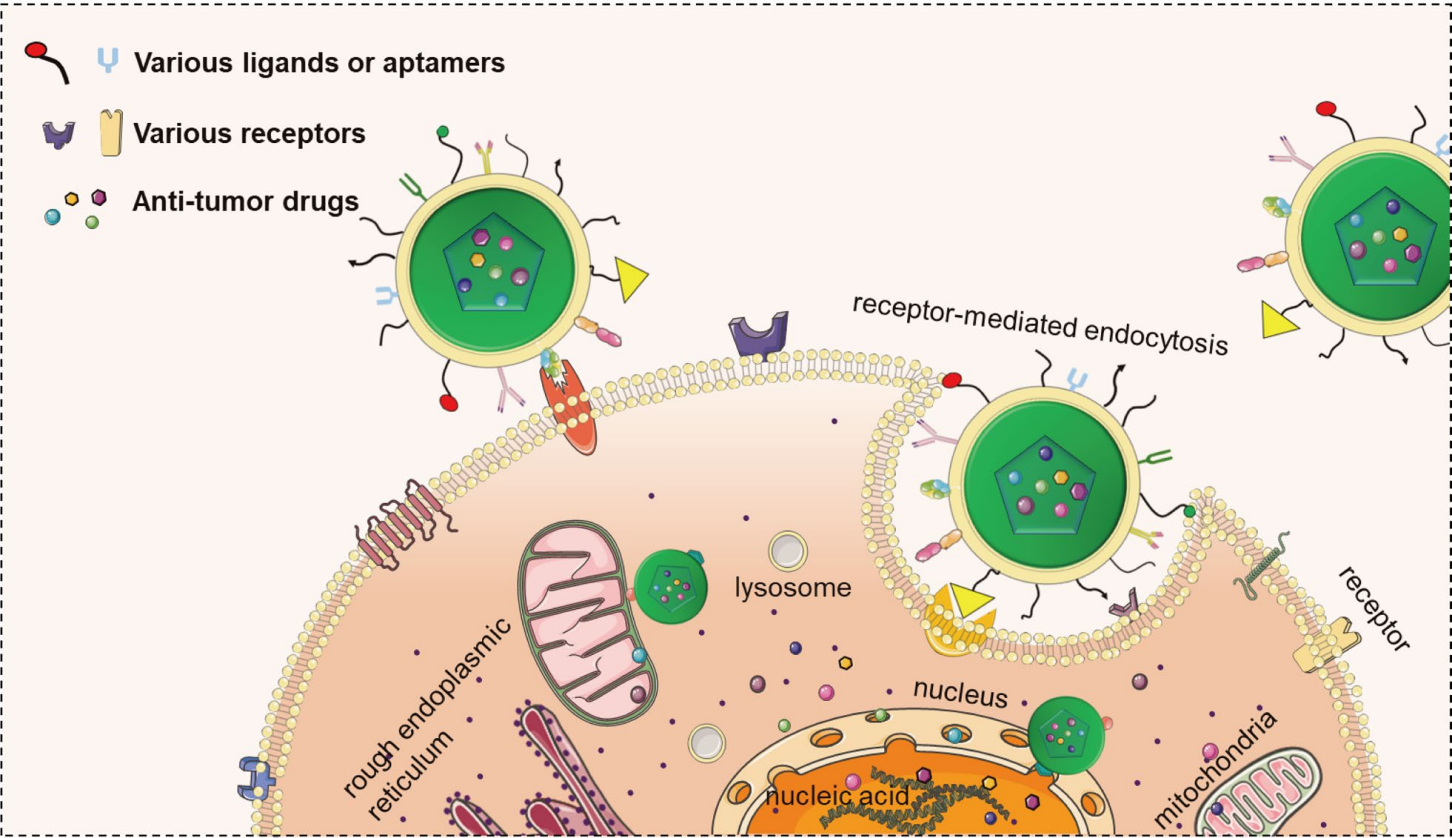
A schematic illustration of osteosarcoma cell internalization nanocarriers by active targeting. Various targeting ligand-modified nanocarriers initiate receptor-mediated endocytosis by binding to receptors on the surface of osteosarcoma cell. In addition to binding to osteosarcoma cell surface markers, nanocarriers can also exert anti-osteosarcoma activity by targeting moiety, such as targeting mitochondria or the nucleus

Various biomarkers are specifically expressed or overexpressed on the tumor cell surface, such as prostate specific membrane antigen (PSMA), which is overexpressed in most prostate cancer cells, and human epidermal growth factor receptor-2 (HER2), which is overexpressed in up to 30% of breast cancers [80]. Active cellular targeting of nano-drug delivery systems is achieved by modifying high-affinity ligands or aptamers to target tumor cells and deliver antitumor drugs [81] (Fig. 4). Aptamers targeting PSMA can be used to target bone metastatic prostate cancer [82]. Trastuzumab (a monoclonal antibody against HER2, TRA) exerts antitumor activity by specifically binding to HER2 in breast cancer cells [80, 83]. Currently, biomarkers such as VEGFRs, integrin, and folate receptors have been selected as targets for the development of OS nano-drug delivery systems. Based on the biological functions and properties of biomarkers and ligands, we classified these targeting schemes into angiogenesis-related active targeting, cell proliferation-related active targeting, Glycan binding proteins-related active targeting, peptide-related active targeting, cancer stem cell–related active targeting, and targeting moiety (Table 2).

**Table 2:** Examples of osteosarcoma cells targeting

Targeting ligand	Nanocarrier type	Agent	Cell lines	References
Ligand CD80 and VEGF Antibody	NPs	Magnetic Iron Oxide	Rodent OSA cell line (ATCCTMNPO CRL-2836)	[98]
VEGF	NPs	Au &DOX	MG63‑Luc cells	[101]
RGD	NPs	AIBI&MnO2&TPP	MNNG/HOS OS cells	[115]
MTX	NPs	MTX & Zn^2+^	Saos-2 OS cell, MCF-7 and T47D human breast cancer cells	[129]
Folate	Nanotubes	MTX	CT26WT murine colorectal cancer, K7M2-WT OS cells and MC3T3-E1 pre-osteoblast cells	[121]
EGFR aptamer	NPs	Salinomycin	U2OS and MG63 OS cells	[135]
Trastuzumab	NPs	Trastuzumab & GO	MG63, HOS and 143B OS cells	[144]
Mannose	Nanocomposite	CD-siRNA	U2OS OS cells	[153]
2DG	Nanocomplexes	GQD	hFOB 1.19 osteoblastic cells and 143B OS cells	[156]
Fructose	NPs	Mn^2+^	143B OS cells and MC3T3-E1 cells	[160]
HA & ALN	Micelles	Curcumin	MG-63 OS cells and human osteoblast cells	[167]
HA	NPs	CDDP&DOX&R848	K7M2 OS cells Bone marrow-derived DCs and bone marrow-derived macrophages	[166]
YSA-peptide	Liposomes	DOX	Saos-2 OS cells and primary bone cells	[176]
STP	Nanogel	Shikonin	hFOB1.19 osteoblast cells and 143B OS cells	[179]
peptide PT	NPs	Semiconducting polymer (PCPDTBT)	4T1 murine mammary carcinoma cells, 143B, MG63 OS cells	[189]
OTP	Nanodiscs	Nanodiscs	HOS OS cells, HUVECs, MLO-Y4, and MC-3T3-E1 cells	[192]
CD133 aptamers	NPs	Salinomycin	Saos-2, U-2 and MG-63 OS cells	[15]
CD271 monoclonal antibody	Nanospheres	HGNs	Saos2 and MNNG/HOS OS cells	[207]
HOS CMs	NPs	IR780	HOS, MG63, 143B and K7M2 OS cells; 4T1 breast cancer cells and A549 non-small-cell lung cancer cells	[216]
WELL5 CMs	NPs	MTX & FUDR	WELL5 and MG63 OS cells; HUVEC cells	[217]
TPP	Nanocomposites	ICG	MG63 OS cells	[229]
NLS peptides	NPs	DOX	Saos-2 OS cells	[231]
CPP&NLS peptides	NPs	Au	143B OS cells	[234]

*VEGF* vascular endothelial growth factor, *RGD* Arg-Gly-Asp, *DOX* doxorubicin, *MTX* methotrexate, *EGFR* epidermal growth factor receptor, *GO* graphene oxide, *2DG* 2-deoxy-D-glucose, *GQD* graphene quantum dots, *HA* hyaluronic acid, *ALN* alendronate, *CDDP* cisplatin, *STP* sarcoma-targeting peptide (peptide VATANST), *OTP* OS targeting peptide, *HGNs* hollow gold nanospheres, *FUDR* floxuridine, *CM* cell membrane, *ICG* indocyanine green, *OS* osteosarcoma, *NLS* nuclear localization signal, *CPP* cell-penetrating peptide

#### Angiogenesis-related active targeting

Targeted angiogenesis has become a major focus area in cancer therapy. New blood vessel development is crucial for solid tumor growth, invasion, and metastasis [84, 85]. Tumor growth requires an adequate blood supply, and anti-angiogenesis and blocking of the blood supply can regulate tumor size and metastatic capacity [86]. Tumor cells secrete various pro-angiogenic factors during the induction of angiogenesis, including vascular endothelial growth factor (VEGF), platelet-derived growth factor, basic fibroblast growth factor, transforming growth factor-α (TGF-α), and TGF-β [87]. In addition, several specific receptors associated with angiogenesis are overexpressed on the surface of tumor cells, including VEGFRs, αvβ3 integrins, matrix metalloproteinases, and vascular cell adhesion molecule-1 [88, 89]. Several studies have developed various ligand-modified nano-drug delivery vehicles based on angiogenic targeting schemes.

##### VEGFR targeting

VEGF is considered the most relevant inducer of tumor angiogenesis. Studies have reported that VEGF expression is negatively correlated with the prognosis of patients with OS [85]. The VEGFR of interest for targeted nano-drug delivery systems is VEGFR-2 because it is interacted with VEGF and is highly expressed on endothelial cells of the tumor neointimal system. VEGFR-2 is tyrosine-phosphorylated upon ligand binding and initiates the signaling cascade response of angiogenesis [90]. Previous studies have shown that VEGFR is expressed in most OS cell lines and primary and metastatic OS [89, 91, 92]. Tyrosine kinase inhibitors of VEGFR-2 such as sorafenib and everolimus approved by the United States Food and Drug Administration (FDA) have been administered for the treatment of OS and have achieved satisfactory efficacy [93–95].

**Fig. 5:**
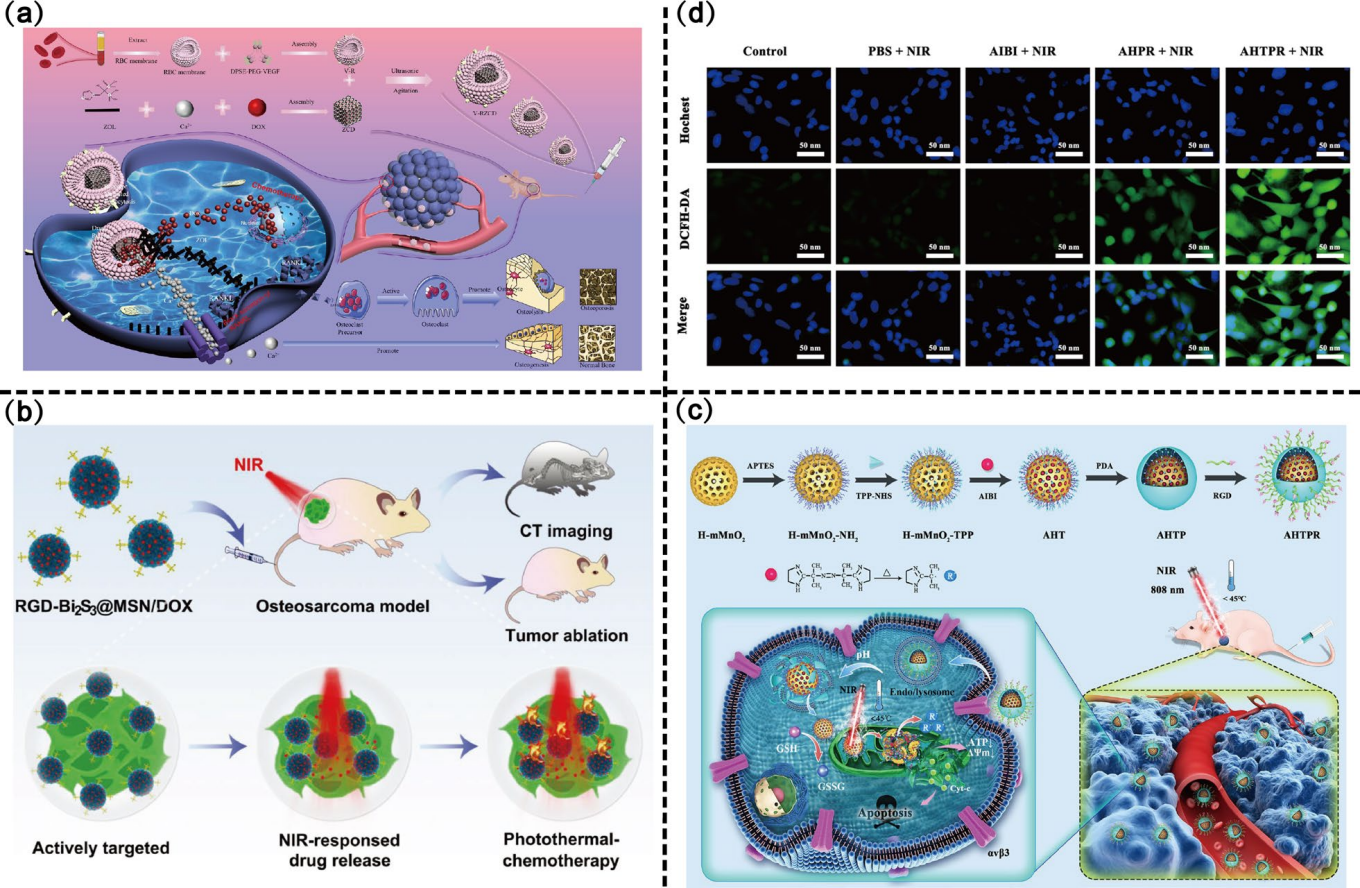
Angiogenesis-related active targeting scheme design of nano-drug delivery systems. **a** Schematic diagram of V-RZCD construction and its targeted application in anti-osteosarcoma and anti-osteolysis. Copyright 2021, ACS Applied Materials & Interfaces. **b** Schematic diagram of the smart RGD–Bi_2_S_3_@MSN/DOX nanoplatform for OS real-time X-ray CT imaging and NIR-responsive photothermal therapy–chemotherapy. Copyright 2018, Advanced Healthcare Materials. **c** Schematic illustration of the synthesis procedures of AHTPR NPs and the application of the NPs in the synergistic mitochondria-targeted low-temperature PTT/ thermodynamic therapy. **d** The generation of alkyl radicals in MNNG/HOS cells after corresponding treatment. Copyright 2021, Journal of Nanobiotechnology

Research on nano-drug delivery systems has explored two main strategies to target angiogenesis via VEGF and VEGFR-2: targeting VEGFR-2 to reduce binding to VEGF and induce the endocytic pathway, and targeting VEGF to inhibit ligand binding to VEGFR-2 [96, 97]. Targeting VEGFR-2 is the main direction of current research. Targeting VEGFR has been attempted in the delivery of proteins, metal ions, bisphosphonates, etc. Kovach, et al. [98] performed dual targeting modification of magnetic NPs with VEGF antibodies and CD80, a ligand for cytotoxic T lymphocyte-associated antigen-4 (CTLA-4). It was reported that CTLA-4 was expressed in human OS, and immune response-induced apoptosis is triggered when ligand CD80 interacts with the CTLA-4 receptor [99, 100]. The combination of CD80 and VEGF significantly reduced the proliferation of aberrant osteoblasts through synergistic targeting, and 1 μg/mL of CD80 + VEGF could be used as the optimal concentration to induce maximal osteoblast proliferation arrest and cell death in rodent OS cells in vitro. Dual-targeted and combination therapies mediated by VEGF have also been developed. Au@SiO_2_-indocyanine green (ICG)/VEGF NPs modified by VEGF achieve a strong synergistic antitumor effect of PTT and chemotherapy, and the tumor growth was almost entirely inhibited on day 25 in Au@SiO2‑DOX/VEGF group [101]. As shown in Fig. 5a, the multifunctional nano-drug delivery system V-RZCD (VEGF-modified erythrocyte membrane nanovesicles) loaded with ZOL and DOX significantly inhibited OS proliferation by targeting the highly expressed VEGFRs [102]. Furthermore, the delivery of BPs significantly inhibited OS-induced osteolysis and preserved more bone structure.

Targeting VEGFRs provides a direction for the treatment of OS, but current studies have assessed the therapeutic effect on OS cells more than the angiogenic inhibition effect by targeting VEGFRs. Therefore, more studies should be conducted to assess the angiogenic inhibition of OS.

##### Integrin targeting

Integrins are a class of pro-isosteric cell adhesion molecules that primarily mediate intercellular and extracellular matrix interactions. Integrins are overexpressed in various tumor cells, such as prostate cancer, gastric cancer, breast cancer, and pancreatic cancer, and are involved in tumorigenesis and progression [88, 103–105]. Integrin αvβ3 is an endothelial cell receptor that is highly expressed in neovascular endothelial cells, which activates calcium-dependent signaling pathways for endothelial cell migration and mediates endothelial cell–ECM interactions [106].

Arg-Gly-Asp (RGD) is a highly conserved recognition motif in ECM proteins that targets certain integrins [107, 108]. In addition, RGD has the advantage of low toxicity and ease of synthesis [109]. Proteolytic cleavage of integrin αvβ3 upon binding to RGD exposes the cryptic CendR motif, which interacts with the neuropeptide-1 receptor that triggers tissue penetration [110]. Integrin αvβ3 is overexpressed in both the tumor vascular system and tumor cells, which provides great potential for targeted tumor therapy [111]. As a well-known cell-homing peptide, the selective recognition of RGD on tumor cells in relation to its internalization ability has made this peptide a promising ligand candidate.

RGD-modified nano-drug delivery systems have demonstrated good therapeutic efficacy in both targeted chemotherapy, phototherapy and gene therapy. Novel RGD-modified OS-targeting DOX-loaded polymeric micelles (RGD-DOX-PM) with high drug-loading efficiency (57%–73%) [112]. Owing to the affinity of RGD and MG-63 membrane integrins, RGD-DOX-PM demonstrated the ability to target and kill OS cells in vitro. Compared with cells pretreated with free RGD, MG-63 cells without free RGD pretreatment showed significantly higher cellular uptake and an approximately sixfold higher ability to inhibit cell proliferation after the addition of RGD-DOX-PM. Thus, RGD-mediated synergistic therapies are promising. Similarly, RGD-mediated active targeting is a prerequisite for Bi_2_S_3_@MSNs to exert excellent photothermal effects, and synergistic DOX-targeted chemotherapy achieves a significant OS-killing ability [113] (Fig. 5b). During the gene therapy of OS, engineered exosomes modified by cyclic RGD peptide (cRGD-Exo-MEG3) demonstrated 2.5-fold tumor targeting ability and twofold antitumor effects in vivo compared with Exo-MEG3 [114].

AIBI@H-mMnO_2_-TPP@PDA-RGD (AHTPR) NPs designed by co-integrating RGD and the mitochondria-targeting ligand (4-carboxybutyl) triphenyl phosphonium bromide (TPP) overcomes the limitations of single targeting [115]. Surface-modified RGD promotes the targeting of AHTPR NPs to OS cells and induces giant cell drinking and lattice-protein-mediated endocytosis (Fig. 5c). After the PDA shell is disrupted in the acidic environment of tumors, NPs achieve targeted accumulation in the mitochondria via TPP. More importantly, intracellular glutathione can be oxidized to glutathione disulfide by MnO_2_, which significantly reduces the consumption of alkyl radicals induced by 2,2'-azobis[2-(2-imidazolin-2-yl) propane] dihydrochloride (AIBI), (Fig. 5d). As a result, several free radicals accumulate rapidly in the mitochondria, lowering the mitochondrial membrane potential and eventually triggering apoptosis in tumor cells [116, 117].

The research found that the RGD sequences have strict targeting specificity for integrins. The affinity of cRGDyk (cyclo (Arg-Gly-Asp-d-Tyr-Lys)) for αvβ5 integrins, which are mainly overexpressed in HCT-116 cells, has been reported to be much weaker than that for αvβ3 integrins. By contrast, cRGDfC (cyclo (Arg-Gly-Asp-d-Phe-Cys)) has a high targeting affinity for αvβ5 integrins [19]. Therefore, when selecting RGD as a targeting ligand, researchers need to evaluate the type of integrin expressed on the OS surface carefully to improve delivery efficiency.

#### Cell proliferation-related active targeting

One of the major features of tumors is uncontrolled cell proliferation [118]. Inhibiting the uncontrolled proliferation of tumor cells is the focus of antitumor therapy, and identifying cell proliferation markers are very essential. Recent studies have identified various overexpressed tumor surface receptors that are closely associated with cell proliferation, such as the human endothelial, transferrin, and folate receptors, and the inhibition of cell proliferation through targeted strategies has emerged as a promising area for tumor therapy [119]. For example, TRA exerts antitumor effects by actively targeting HER2 in breast cancer cells. Cell proliferation-related targeting strategies have also been used in OS treatment. Several nano-drug delivery systems modified with folate or HER2 ligands have been developed and have demonstrated good active targeting effects.

##### Folate receptor targeting

Folate, involved in the synthesis of nucleic acids and amino acids, is required for the maintenance of cellular function, especially for infinitely proliferating tumor cells [120]. Thus, cargoes attached to folate ligands are readily retained within endocytic vesicles or released into the cytoplasm. The folate receptor is overexpressed in many cancers, with little or no expression in most normal tissues, which provides a theoretical basis for targeting bone tumors through interaction with the folate receptor [121].

**Fig. 6:**
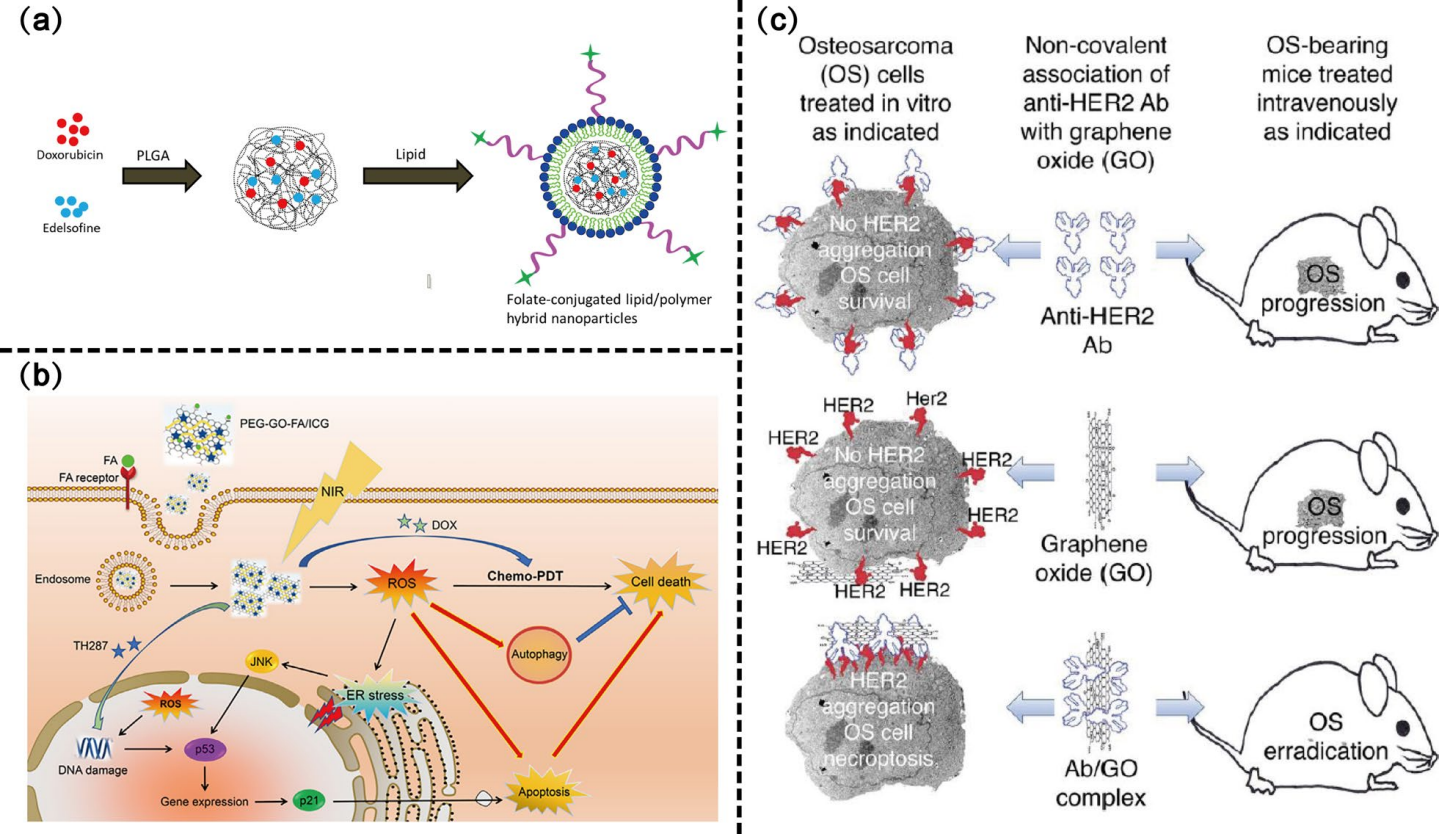
Cell proliferation-related active targeting scheme design of nano-drug delivery systems. **a** Schematic presentation of preparation of DOX and edelsofine-loaded folate targeted lipid–polymer hybrid NPs. Copyright 2020, OncoTargets and Therapy. **b** Overview of Chemo-PDT induced cell death pathways. Copyright 2020, Acta Biomaterialia. **c** Multivalent Ab/GO complexes demonstrate substantially enhanced HER2-binding avidity and capacity to induce HER2 aggregation (capping) on the target osteosarcoma (OS) cells while the free Ab failed to do so. Copyright 2018, Nanomedicine: Nanotechnology, Biology, and Medicine

Various folate-modified nano-drug delivery systems demonstrated good targeting effects with loaded chemotherapeutic drugs such as DOX, curcumin, and salinomycin (SaL), which enhanced the cytotoxicity of OS cells and stem cells [122–124]. Studies have shown that dual-drug combinations are more effective for antitumor treatment. Lipid polymer hybridized NPs loaded with DOX and edelfosine with folate receptor targeting enhanced intracellularization and subcellular distribution in MG63 cancer cells, whereas the co-administration of both chemotherapeutic agents had the significant synergistic ability to induce apoptosis and cell death [125] (Fig. 6a). In addition to delivering the chemotherapeutic agents, folate-modified targeted gene therapy also achieved satisfactory results. As a multifunctional oncoprotein, AEG-1 overexpression was significantly correlated with tumor cell proliferation and invasion, and it is important to consider AEG-1 a potential target for OS gene therapy. Wang et al. [126] designed Cs-g-PLLD-FA/siAEG-1 NPs for the targeted delivery of siRNA (*siAEG-1* gene). SiAEG-1 targeted delivery significantly inhibited 50% OS cell growth and invasion as well as 91% lung nodule development in tumor-bearing mice, which provide a promising delivery system for eradicating OS.

In addition to targeted chemotherapy and gene therapy, folate-modified metal NPs also exerted excellent OS target-killing effects. The modification of folate achieved 99.53% uptake efficiency of metal nanocarriers in 2 h [120]. Oxidative stress is an important pathway to induce tumor cell death for metal NPs, and folate-modified titanium dioxide NPs induced more ROS generation, resulting in higher sub-G0 cell populations and higher cell apoptosis [16, 127]. The magnetic and photothermal properties of metallic nanomaterials have broadened their scope for the treatment of OS. Huang et al. [128] designed polyethylene glycol (PEG)-graphene oxide (GO)-ferulic acid (FA)/ICG NPs to exert chemophotodynamic combination therapy (chemo-PDT), which exerts combined antitumor activity by enhancing ROS production and inducing endoplasmic reticulum stress-induced apoptosis (Fig. 6b).

As a widely studied ligand, the effect of folate-mediated tumor targeting is unquestionable. However, it remains unclear whether cell proliferation is inhibited through folate receptor targeting. Meanwhile, the folate receptor-mediated endocytosis pathway also deserves attention. The basic cellular uptake mechanisms of NPs including phagocytosis, macropinocytosis, the classical lattice protein-mediated pathway and niche-mediated endocytosis, and the transport pathway of NPs may affect the final destination and effectiveness of the drug delivery system [81]. Meshkini et al. [129] investigated the internalization mechanism of MTX-F127@ZnHAP NPs by using cellular uptake route inhibitors, which found that lattice-protein-mediated endocytosis was the main internalization mode. Furthermore, once the lattice protein-mediated pathway was blocked, fossa-mediated endocytosis serves as an alternative pathway.

##### Human endothelial receptor targeting

Human endothelial receptor is a class of transmembrane tyrosine kinase receptors with four members. Among them, epidermal growth factor receptors (EGFR) and HER2 are closely associated with cell proliferation and have been studied extensively in tumors [130]. EGFR and HER2 have great potential as specific targets for nano-drug delivery systems for OS treatment.

EGFR has multiple endogenous ligands, including EGF and TGF-α, which enable possible EGFR as a targeting fraction [130]. Ligand binding to EGFR leads to the activation of intracellular signaling cascades. Small-molecule inhibitors (e.g., erlotinib) and antibodies (e.g., cetuximab) targeting EGFR achieve superior therapeutic efficacy in oncology treatment [131]. EGFR mutations and gene amplification are characteristic genetic abnormalities detected in OS. Furthermore, the elimination of EGFR phosphorylation in OS inhibits growth, whereas EGFR overexpression promotes tumor cell invasion [132]. Phase I clinical trials of EGFR inhibitors for the treatment of OS are currently underway [133, 134]. EGFR is an important target for OS treatment, and studies have confirmed its high expression in various OS cell lines and stem cells. EGFR aptamer–coupled EGFR-SNPs were designed to efficiently deliver SaL, enhancing OS cytotoxicity, inhibiting tumor sphere formation, and reducing the proportion of CD133 + OS stem cells (OSCs) [135]. OSCs are closely associated with tumor drug resistance, and targeting tumor stem cells may improve tumor treatment efficacy. Dual-target ligand modifications have also demonstrated excellent therapeutic potential. As a specific marker on the surface of OSCs, targeting CD133 can effectively eliminate CD133 + OSCs. By co-modified with the CD133 + ligand (A15) and EGFR aptamer (CL4), CECP NPs achieved dual targeting effects of OSCs and OS cells, which reduced fourfold tumor spheres formation and the percentage of CD133 + cells, and obtained a 90% decrease of tumor volume in vivo [136].

HER2-driven tumorigenesis reportedly results from HER2 protein overexpression on the cell surface due to *HER2* gene amplification, which promotes HER2 homo- or heterodimerization and activates HER2 tyrosine kinases [137]. Sustained HER2 activation and downstream signaling constitute strong oncogenic stimuli. Given the presence of HER2 overexpression in multiple tumors, the FDA approved the use of anti-HER2 antibodies (TRA and patuximab) for the treatment of HER2 + tumors (e.g. breast and gastroesophageal cancers), which significantly prolonged the survival and improved the prognosis of patients [138]. Approximately 40–60% of OS overexpress HER2, but usually, no *HER2* gene amplification occurs [138–140]. A low level of HER2 overexpression indicates that HER2 may not play an important role in the carcinogenesis of OS [141]. Studies have confirmed no significant inhibition when treating OS cell lines with TRA [142]. Phase II clinical trials of TRA to treat HER2 + metastatic OS also did not observe a therapeutic benefit [143]. However, HER2-mediated targeted nano-drug delivery systems have shown promising results in the treatment of OS. The non-covalent association of graphene oxide with TRA increased TRA responsiveness and affinity for HER2 (Fig. 6c). The targeted antitumor activity of TRA–GO complexes is achieved through two aspects: HER2-mediated cytotoxicity and GO-induced oxidative stress and ROS production, which rapidly depletes cIAPs and caspase 8 and induces cancer cell death via necrosis [144]. In addition, satisfactory anti-OS efficacy was achieved using HER2-specific CAR-T cells. Culturing HER2 OS cells with HER2-specific CAR-T cells expressing the transgenic CD28-zeta structural domain stimulated a strong Th1 response and produced cytotoxicity against target OS cells [142]. Overall, HER2-mediated OS-targeted therapy still needs to be supported by more studies.

#### Glycan binding proteins-related active targeting

Studies have demonstrated that carbohydrates play a key role in cellular recognition processes such as enzyme transport, cell migration, cancer metastasis, and immune function [145, 146]. Compared with normal cells, tumor cells have a higher demand for nutrients because of rapid proliferation capacity. Increased glycolysis (i.e., the Warburg effect) and high demand for pentose (e.g., glucose and fructose) are common metabolic features of tumor cells [147, 148]. The overexpression of various proteins that can bind to sugars was identified on the surface of tumor cells, implying that glycans or polysaccharides could likewise serve as potential ligands for targeted drug delivery [149].

##### Lectin receptor targeting

**Fig. 7:**
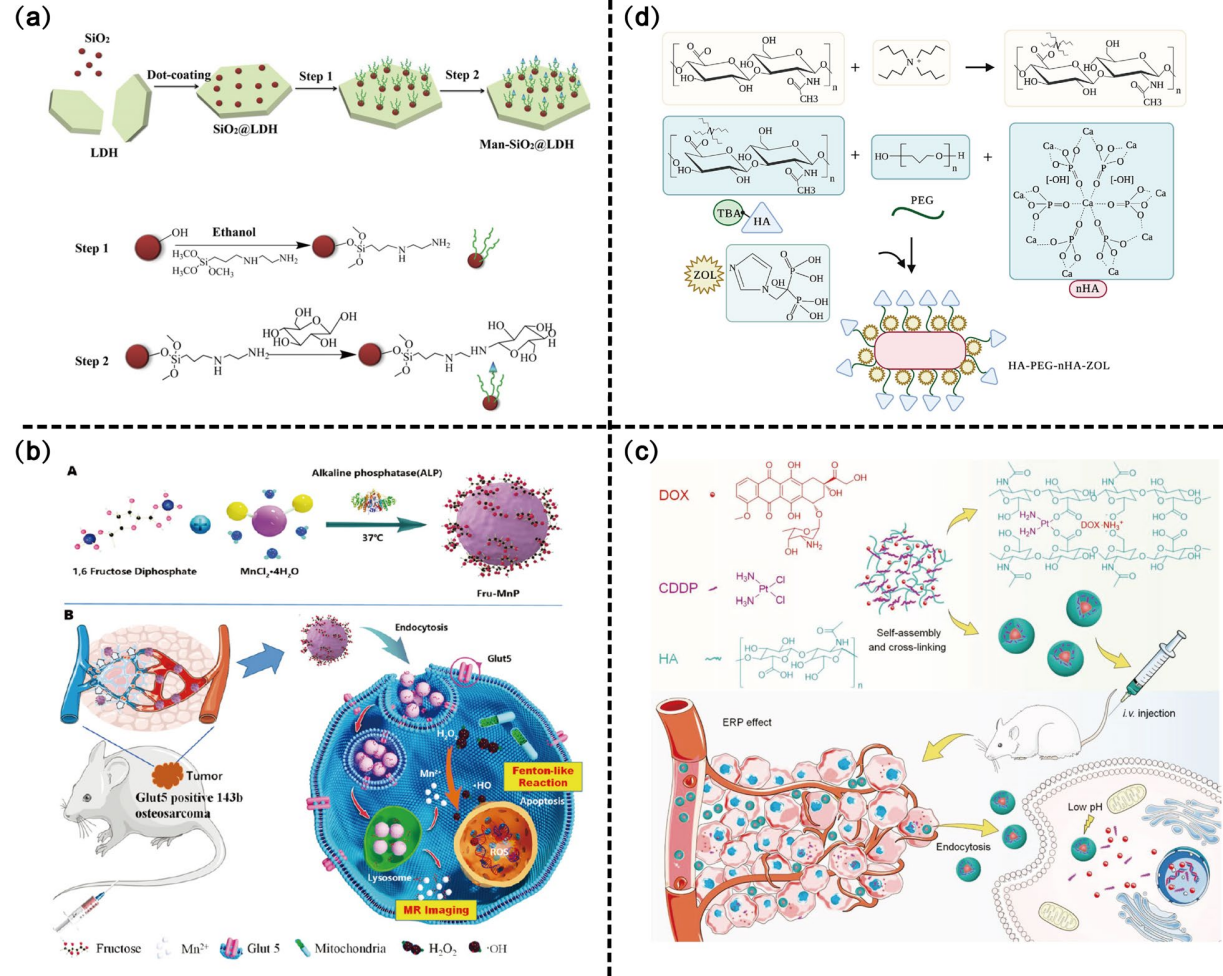
Glycan binding proteins-related active targeting scheme design of nano-drug delivery systems. **a** The synthetic process of Man-SiO2@LDH nanocomposite. Copyright 2017, Nanomedicine: Nanotechnology, Biology and Medicine. **b** Schematic illustration of Fru–MnP nanocomposites prepared from fructose-1,6-diphosphate (FDP) and MnCl_2_·4H2O for the catalytic therapy of osteosarcoma. Copyright 2022, Nanoscale. **c** Schematic illustration for preparation, intravenous injection, in vivo circulation, selective accumulation in tumor tissue, and pH-triggered intracellular drug release of ^CDDP^HANG/DOX. Copyright 2018, Advanced Science. **d** Schematic diagram of the construction of HA-PEG-nHA-ZOL nanoparticles. Copyright 2022, Frontiers in Bioengineering and Biotechnology

Mannose displays a high binding affinity for lectin receptors, which are highly expressed in cancer cells [150]. Mannosylated NPs were recently developed to increase drug accumulation, particularly in tumor cells overexpressing lectins [151, 152]. Mannose-coupled lamellar layered double hydroxide (LDH) nanocomposites (man-SiO_2_@LDH) were designed for targeting delivery of siRNA [153] (Fig. 7a). Cellular uptake showed that mannose modification resulted in the efficient uptake of human SiO_2_@LDH by U2OS cells through lectin receptor-mediated endocytosis. When the lectin receptor was blocked by free mannose molecules, the receptor-mediated endocytosis was blocked, which implies that mannose-mediated targeting delivery is specific. In terms of cytotoxicity, Man-SiO_2_@LDH exhibited 66% U2OS cell-killing and a 2.5-fold improvement in tumor cell growth inhibition compared to LDH without mannose modification.

##### Glucose transporter proteins targeting

Targeting glucose transporter proteins on the surfaces of tumor cells is another promising targeted therapy strategy. 2-Amino-2-deoxy-d-glucose (2DG) increases glucose consumption by tumor cells by targeting glucose transporter proteins [154, 155]. Tung et al. [156] grafted 2DG onto nonmetallic graphene quantum dots (GQD) to achieve radiosensitization effects on primary and metastatic OS. Furthermore, 2DG-g-GQD impaired the migration ability of OS cells in a way that restrained lamellipodia and filopodia formation and deranged F-actin filaments, further reducing distant lung metastases by 95% compared with radiation therapy alone. However, whether 2DG-g-GQD affects the glucose metabolic process of OS cells by targeting glucose transporter still needs further evaluation.

Fructose can replace glucose in tumor cells for glycolysis. Enhanced fructose utilization and high expression of the fructose transmembrane transporter protein (GLUT5) are prevalent in tumor cells [157–159]. GLUT5 can be used as a marker to distinguish OS from normal tissues and is a promising target for future OS therapy. Zhang et al. [160] prepared Fru-MnP nanoplatform by an alkaline phosphatase (ALP)-catalysed route. Fructose strongly interacts with GLUT5 overexpressed on CMs and is selectively taken up by OS cells through receptor-mediated endocytosis. Mn^2+^ ions were released from internalized Fru-MnP and catalyzed endogenous H_2_O_2_ into hydroxyl radicals (•OH) via a Fenton-like reaction, triggering chemodynamic therapy (Fig. 7b). Compared with GLUT5-negative MC3T3-E1 cells, Fru-MnP was more selectively internalized by GLUT5-positive 143B cells, significantly inducing cell death, whereas co-culture of Fru-MnP with GLUT5-negative MC3T3-E1 cells barely affected cellular activity (cell viability: 24 h, 98%; 48 h, 87%).

##### CD44 targeting

HA is a glycosaminoglycan composed of *N*-acetyl-d-glucosamine and d-glucuronide, which is distributed in the ECM and has excellent biocompatibility and biodegradability [161]. The HA receptor CD44 is overexpressed by many tumor cells, and the specific binding of HA to CD44 can increase tumor site aggregation and achieve accurate targeting of certain cancer cells [162–165].

HA has been extensively developed as a targeting ligand for nano-drug delivery systems in active targeting strategies for OS. As shown in Fig. 7c, CDDP-cross-linked HA nanogel (^CDDP^HANG) enables targeted chemotherapy of OS by delivering DOX. The ^CDDP^HANG/DOX delivery platform significantly prolonged the retention time and enhanced anticancer efficacy [10]. The HA-modified multidrug delivery system (^CDDP^NP_DOX&R848_) induced apoptosis and immunogenic cell death, greatly inhibiting tumor growth and lung metastasis [166]. The targeted delivery of BPs modified by HA effectively inhibited the in situ recurrences of OS along with antitumor effects [167–169]. Xu et al. [168] constructed an HA-modified polyethylene glycol/nano-hydroxyapatite (HA-PEG-nHA) NPs system for ZOL delivery** (**Fig. 7d). HA binds to CD44 in OS cells, which provides a prerequisite for the therapeutic effects of ZOL. In addition, the acid-sensitive separation of PEG allows NPs to achieve targeted drug release in the special acidic environment of the tumor, which greatly improves the utilization efficiency of ZOL. Moreover, nHA rescues tumor-induced osteolysis and plays an active role in bone repair after bone tumor resection.

Targeted nano-drug delivery systems based on glycans appear to be promising delivery options. However, sugars like fructose are one of the main energy sources for bone tumor cells, so it remains to be further verified whether such targeted ligand-modified delivery systems will provide energy in disguise and promote bone tumor cell proliferation.

#### Peptide-related active targeting

Targeting peptides have an excellent binding affinity and specificity for extracellular ligands, which have the advantages of easy synthesis, good histocompatibility, low immunogenicity, and good pharmacokinetics and have become a candidate for molecular targeting ligands [170, 171]. Targeting peptide-modified nano-drug delivery systems can be a new active targeting strategy. NPs modified with targeting peptides could greatly improve tumor-targeting ability [171]. Currently, various specific tumor-targeting peptides had been developed and applied in drug delivery.

##### YSA targeting

**Fig. 8:**
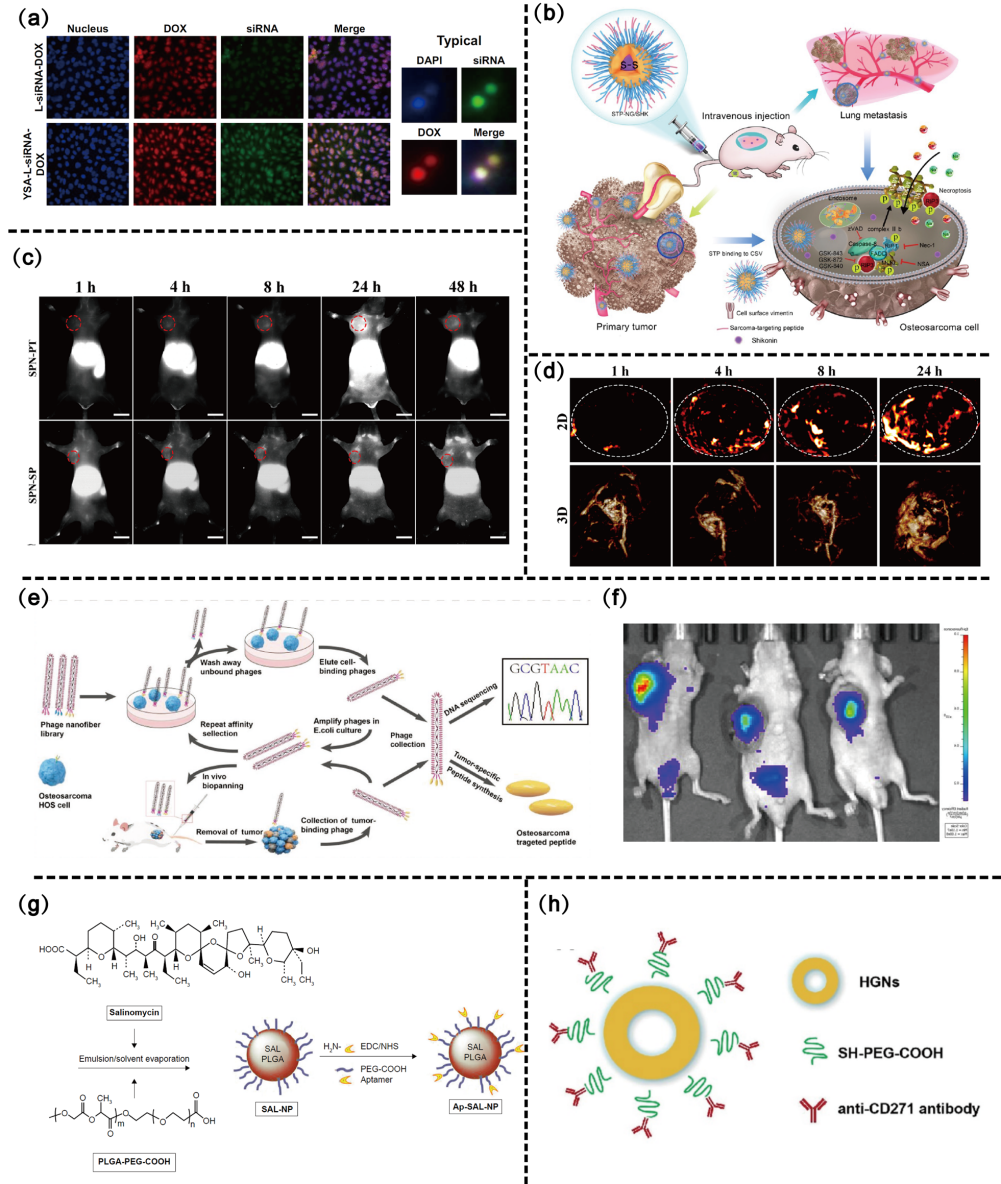
Peptide-related and cancer stem cell-related active targeting scheme design of nano-drug delivery systems. **a** Comparison between cellular uptake of targeted and nontargeted formulations. Copyright 2018, International journal of nanomedicine. **b** Schematic illustration for preparation of STP-NG/SHK, and RIP1- and RIP3-dependent cell necroptosis in primary tumor and lung metastasis. Copyright 2018, Theranostics. **c** NIR-II fluorescence images of 143B xenograft mice at different time points post injection of SPN-PT or SPN-SP. Scale bar = 1 cm. **d** Two-dimensional (upper) and three-dimensional (lower) PA imaging of 143B tumors in living mice injected with SPN-PT at different time points. Copyright 2022, Journal of nanobiotechnology. **e** Schematic description of evolutionary selection of osteosarcoma HOS cell/tissue dual-homing phage nanofibers from a phage library by a combination of in vitro and in vivo biopanning against osteosarcoma cells and tumor tissues. **f** Living image show the tumor targeting and retention of two-dimensional nanodiscs after 24 days of injection. Copyright 2022, Small. **g** The preparation procedure of SAL-NP or Ap-SAL-NP. Copyright 2015, International Journal of Nanomedicine. **h** Schematic illustration of anti-CD271 antibody conjugated HGNs. Copyright 2020, Nanotechnology

YSA (YSAYPDSVPMMS) is a 12-amino-acid peptide that is a mimetic of epinephrine A1 and a ligand for the epinephrine alpha 2 receptor (EphA2) [172–174]. As a surface molecule, EphA2 is highly expressed in primary and metastatic OS cells. Studies to date focused on the high affinity of YSA for EphA2 for the targeted delivery of chemotherapy drugs [175]. Haghiralsadat et al. [176] prepared YSA-l-DOX NPs by covalently binding the YSA peptide to polyethylene glycolized liposomes with a very high coupling efficiency (98%) without affecting their properties. Moreover, owing to the modification of the YSA peptide, the uptake of DOX-loaded liposomes by Saos-2 OS cells was promoted, and cytotoxicity was increased by nearly twofold. However, the present study did not evaluate its in vivo targeting effect. To overcome multidrug resistance in OS, dual targeting of DOX-loaded liposomes (extracellular YSA peptide–mediated targeting against the upregulated surface marker EphA2 of OS and intracellular siRNA against JIP1 [JNK-interacting protein 1] to improve chemosensitivity) has been designed [177]. After the YSA modification, YSA-L-siRNA-DOX achieved more substantial transfection than L-siRNA-DOX, which increased the cell-killing rate of OS cells by 1.5-fold and may effectively treat metastatic OS (Fig. 8a). Thus, the YSA peptide may be the superior targeting ligand in targeted nano-drug delivery in metastatic OS.

##### STP and PT targeting

In addition to YSA, several specific tumor-targeting peptides have been developed. The peptide VATANST (STP), which binds specifically to the waveform protein vimentin, is highly expressed on the surface of OS cells and is strongly associated with tumor metastasis [178–182]. The targeted peptide-modified STP-NPs (the STP-decorated mPEG-P(Phe-co-Cys) NPs) specifically bind to waveform proteins and enhance the DOX transport ability of CMs, thereby improving the efficiency of tumor suppression in OS and reducing the side effects of DOX on the major organs [183].

Similarly, Li et al. [179] prepared STP-modified reduction–responsive PEG-poly (l-phenylalanine-co-l cytosine) (STP-PEG-P(LP-co-LC)) nanogels for the targeted intracellular delivery of shikonin (STP-NG/SHK) (Fig. 8b). Owing to tumor-specific targeting, STP-NG/SHK showed superior antitumor efficacy against SHK and NG/SHK in OS and enhanced cell necrosis via the RIP1 and RIP3 pathways. Most importantly, STP-NG/SHK treatment reduced pulmonary metastasis, which achieved 71.4% in the 90-day survival rates and ultimately increase long-term survival. Targeting OS cells via STP provides new inspiration for active targeting nano-drug delivery.

Semiconductor polymer NPs (SPN) are an emerging class of organic photonic agents fabricated from optically active semiconductor polymers, which are widely used in fluorescence imaging, photoacoustic imaging, PTT and other fields [184–187]. Peptide-modified semiconductor NPs have shown promising phototherapeutic effects. Studies reported that peptide PT (PPSHTPT) mimics the natural protein osteocalcin and shows targeted binding to OS in vitro and in vivo [188]. Yuan et al. [189] developed a SPN based on oligopeptide PT (SPN-PT), which showed an OS-targeting ability. Due to the PT peptide, SPNs can be actively internalized into OS cells within 4 h, enhancing NIR-II fluorescence and PA signals, and thus providing high-sensitivity and high-resolution images and promoting phototherapy efficacy (Fig. 8c, d). In addition to mediating target binding, it is worth investigating whether targeting peptides can induce apoptosis or inhibit the proliferative capacity of OS cells.

##### Tumor-targeting peptides targeting

Despite the improved tumor-targeting capability of nanocarriers, generic targeting peptides cannot be used to target highly heterogeneous cancers because of the ease of off-targeting and susceptibility to the expression levels of cellular receptors on tumor cells [190, 191]. For highly heterogeneous cancers, phage display in tumor cells and tissues to identify specific targeting peptides could address the lack of effective tumor-specific binding ligands.

To achieve tumor targeting and prolong retention in highly heterogeneous bone tumors, Lin et al. [192] screened highly specific tumor-targeting peptides with the sequence of TPPPRVPLLTFGS using multi-round phage display technology (Fig. 8e). Two-dimensional nanodiscs modified by specific tumor-targeting peptides not only exhibited excellent heterogeneous tumor-targeting properties and promoted the uptake of nanodiscs by tumor cells, they significantly prolonged tumor site retention for up to 24 days, much longer than ND and other NPs (typically < 72 h) [193] (Fig. 8f). After a single intravenous injection into tumor-bearing mice, 2-dimensional nanodiscs can act as a tumor-tailored photothermal agent (PTA), resulting in PTT that precisely destroys the tumor. It also largely alleviates PTT side effects due to off-target delivery and shows great potential for precise PTT in highly mutated cancers. This offers the possibility of personalized targeted drug delivery in clinical practice.

Targeting peptides are favored by researchers because of their excellent biocompatibility and low immunogenicity, and several studies have confirmed the attractive target binding ability. Huge histological heterogeneity and genomic instability of OS remain a major challenge for current drug delivery, and more specific targeting peptides should be developed for individualized treatment of OS.

#### Cancer stem cell-related active targeting

Cancer resistance, recurrence, and metastasis are the main causes of cancer treatment failure. Cancer stem cells (CSCs), the primary cancer-initiating cells, are more resistant to conventional therapy than normal cancer cells and play an important role in processes of metastasis and recurrence [194]. Researchers have found OSCs have self-renewal potential and the new tumor-generation ability, and conventional therapies that induce apoptosis in non-OSCs have little effect on OSCs [195–199]. OSCs represent a new target for OS therapy, and innovative therapies targeting CSCs and normal tumor cells may more effectively eradicate cancer.

##### CD133 targeting

Previous studies demonstrated that CD133 + OS cells exhibit more stem cell characteristics, including low abundance, quiescence, high differentiation potential, and expression of stem cell regulatory and drug resistance genes [200–202]. Specific killing of OSCs by targeting CD133 + is essential for complete OS eradication.

A15 is an RNA aptamer, which can bind to CD133 + and effectively mediate cellular internalization. It has been successfully used as a targeting ligand to track CD133 + cancer cells and applied in the targeted therapy of OS. SaL (a polyether ion carrier antibiotic isolated from Streptomyces) kills OS-initiating cells by decreasing the tumorsphere formation capacity and percentage of CD133 + cells in OS cell lines. However, the poor water solubility of SaL hinders its clinical application. Based on the properties of A15 targeting CD133 + , Ni et al. [15] developed a PEGylated PLGA NP (Ap-SaL-NP), which improved the aqueous solubility of the SaL (Fig. 8g). It was further demonstrated that the aptamer-modified NPs had specific killing effects on CD113 + Saos-2 cells as well as a targeted effect on OS xenograft mice. Similarly, lipid polymers conjugated with the CD133 aptamer markedly reduced the formation of colonies, inhibited the number of tumoespheres and lowered the percentage of CD133 + OS cells [203].

Continuous delivery of chemotherapeutic agents to OSCs via aptamer-targeted CD133 has unique advantages. As the initiating cells of OS, OSCs are closely associated with multidrug resistance and tumor heterogeneity, and have always been the focus of OS treatment. Serving as a mature biomarker for OSCs, CD133 is an important target for drug delivery. The development of new aptamers to target CD133 + OS cells is a promising therapeutic strategy.

##### CD271 targeting

As a neural crest nerve growth factor receptor, CD271 is a specific biomarker for MCS [204, 205]. CD217 has been found to be expressed in various cancers. In previous studies, CD271 + OS cells had stem cell characteristics such as self-renewal, sphere formation, and drug resistance, suggesting that CD271 could also serve as a potential novel surface marker for OSCs [206]. The HGNs-PEG-CD271 (hollow gold nanospheres, HGNs) nano-drug delivery system interacting with the receptor through the CD271 antibody promotes active uptake and reaches a peak at 24 h (Fig. 8h). Under NIR laser irradiation of 8 W/cm^2^, HGNs-PEG-CD271-mediated PTT induced 12.1% cell viability and DNA double-strand breaks, demonstrating superior anti-bone tumor effects [207].

The treatment of OSCs is important for the eradication of OS, and other biomarkers of OSC, such as CD117 and STRO-1, should be further developed, which would enrich the targeting schemes for OS treatment [208]. In addition to mediating target binding, it remains to be investigated whether aptamers can trigger the death mechanism by cluster of differentiation overexpressed on the surface of OSCs.

#### Targeting moiety

Active targeted nano-drug delivery systems for OS are not limited to the identification of specific biomarkers on the tumor surface. Another important component of the targeted delivery system is the “targeting moiety”, which delivers drugs by targeting organelles or components of the tumor cell. Multiple organelles maintain the normal physiological functions of tumor cells, and disrupting their integrity by destroying normal functioning can be equally lethal to tumor cells. To date, a variety of nano-drug delivery systems targeting CMs, mitochondria and nuclei have been developed and have achieved effective tumor-killing capabilities [209–211].

##### CM targeting

The phospholipid bilayer structure of CMs encapsulates various proteins that are involved in many important biological processes [212]. Moreover, CMs are extremely biocompatible. Nano-drug delivery systems coated with various CM have the properties of the original CM as well as those of nanodrug cores. For example, tumor CM-encapsulated NPs have homologous targeting properties and can adhere to and aggregate in tumor tissues. CMs derived from different types of cells have been modified for targeted drug delivery, for example, VEGF-modified red blood CMs such as nanoshells (V-RZCD) can escape immune recognition [102] and platelet membranes for tumor-targeting NPs in multiple cancer engineering [213, 214]. CM-modified nano-drug delivery systems hold promise as active targeting strategies for OS.

**Fig. 9:**
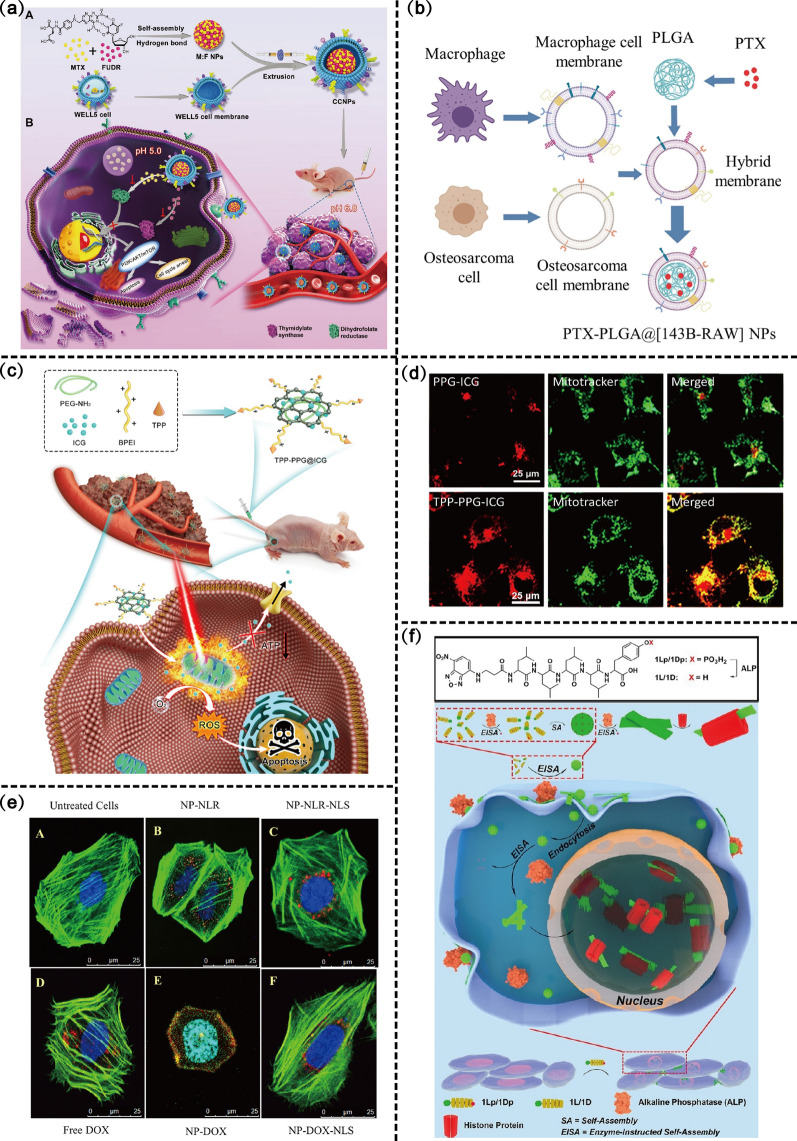
Targeting moiety scheme design of nano-drug delivery systems. **a** Schematic illustration of cancer cell membrane-coated NPs (CCNPs) for OS treatment. Copyright 2022, Small. **b** Preparation of paclitaxel (PTX)-loaded PLGA nanoparticles with 143B-RAW hybrid membrane coating (PTX-PLGA@[143B-RAW] NPs). Copyright 2022, International Journal of Nanomedicine. **c** Schematic illustration showing a TPP-PPG@ICG nanocomposite targeting a mitochondrion for synergistic phototherapy with a single laser. **d** FL-confocal microscopic images of MG63/Dox cells co-treated with MitoTracker and either PPG@ICG or TPP-PPG@ICG are shown. Copyright 2021, Journal of Nanobiotechnology. **e** Confocal microscopy for NLS-mediated nuclear targeted delivery. NPs successfully accumulated around the nucleus. Copyright 2018, Journal of Biomaterials Science, Polymer Edition. **f** Schematic representation of EISA of 1Lp or 1Dp to result in intranuclear assemblies. Copyright 2022, Angewandte Chemie International Edition

Nanodrugs coated with cancer CMs (CCMs) have strong homologous targeting ability and biocompatibility owing to their surface-specific membrane proteins, which may ultimately improve drug delivery efficiency and amplify tumor-killing efficacy. Zhang et al. [215] treated modified silica nanoparticles (SLN) with CM surfaces from 143B OS cells to construct a platform (CM/SLN) that can target homogeneous 143B OS cells. Moreover, ICG was encapsulated into CM/SLN as a PTA to construct CM/SLN/ICG, a drug delivery platform suitable for targeting PTT in OS. In addition to the 143B OS cell-derived CMs, CMs derived from HOS cell line and WELL5 cell line also demonstrated good homologous targeting effects [216]. Fu et al. [217] extracted CMs of WELL5 OS cell line and assembled floxuridine (FUDR) with MTX to form CCM-coated NPs (CCNPs). CCNPs promote cellular uptake and specific accumulation at tumor sites, and ultimately enhances the anticancer activity of MTX (Fig. 9a).

CM derived from a single cell line can only be used for homologous targeting of a single species of tumor cells. To further expand the targeting range, hybrid membranes by integrating two different CMs have been developed. The advantage of hybridized membranes is that the targeting properties of both cell types can be integrated. Cai et al. [218] successfully prepared PTX-loaded PLGA@[143B-RAW] NPs with a drug loading of approximately 4% by mixing CMs derived from the human 143B OS cell line and mouse monocyte-macrophage cell line RAW264.7 (Fig. 9b). Macrophage membrane-modified NPs not only exhibit immune escape properties and enhanced blood retention time but also show chemotaxis to the inflammatory environment [219, 220]. By leveraging the homologous targeting of tumor CMs, PLGA@[143B-RAW] NPs exhibited stronger OS cytotoxicity than unmodified PLGA NPs. The use of macrophage membranes also improved the chemotaxis of NPs to the inflammatory milieu, resulting in a greater preference for sites of inflammation.

Although the dual membrane modification significantly increased the enrichment in tumor tissues, the in vivo biodistribution of NP appeared to be significantly higher in the spleen and liver tissues than in tumor tissues, which is worth considering. Therefore, the CM-modified nano-drug delivery system could provide a good platform for active targeting in OS, but further in-depth studies are needed because of its relatively low drug-loading capacity.

##### Mitochondrial targeting

The mitochondria play a crucial role in various biological processes [221–223]. Alterations in the mitochondria are markers of tumorigenesis, tumor progression, angiogenesis, and chemotherapy resistance [224]. In recent years, mitochondria have received considerable attention as effective targets for tumor therapy. Current various metal NPs have a preference for the mitochondria, such as zinc oxide NPs that accumulate in the mitochondria and exert mitochondrial destructive effects [225, 226]. Special compounds, such as PENAO (4-(*N*-(S-penicillinylacetyl) amino) phenyl arsenate) and poly [2-(*N*-oxide-*N, N*-diethylamino) ethyl methacrylate), also have targeted affinity for the mitochondria and can exert anti-OS efficacy by affecting mitochondrial function [227, 228].

TPP as a mitochondria-targeting ligand has excellent targeting ability and is the most commonly used scheme to specifically disrupt the mitochondria of OS cells. Zeng et al. [229] developed TPP-PPG@ICG NPs and used graphene oxide and ICG as photosensitizers and PTAs to target phototherapy (PDT and PTT) (Fig. 9c). Targeted mitochondrial therapy destroys mitochondrial function, alters mitochondrial membrane potential, reduces ATP production, and induces apoptosis of bone tumor cells (Fig. 9d). To further improve the mitochondrial targeting ability, RGD-modified AIBI@H-mMnO_2_-TPP@PDA-RGD (AHTPR) NPs induced giant cell drinking and lattice protein-mediated endocytosis of OS cells, which provided the prerequisite for subsequent TPP targeting of the mitochondria and the induction of alkyl radical burst [115]. Overall, targeting mitochondria is a scheme with great potential for tumor treatment [230].

##### Nuclear targeting

Similarly, nuclear targeting is another important component of the “targeting moiety” strategy. Chemotherapeutic drugs like DOX exert antitumor effects by entering the cell nucleus and inhibiting the synthesis of genetic material. Therefore, DOX delivery to the perinuclear and intranuclear of tumor cells can maximize their therapeutic effects.

As a specific oligopeptide, the nuclear localization signal (NLS) has good nuclear targeting ability. Current NLS-modified drug delivery systems are making great progress in specific recognition of nuclei and tumor treatment. Targeted delivery of DOX-loaded NPs to the nucleus is achieved under the mediation of NLS, which increases the accumulation of DOX around the nucleus [231] (Fig. 9e). NLS-modified metal NPs also hold great therapeutic potential for nuclear targeting. El-Sayed et al. [232] showed that AuNPs modified with NLS peptides targeted the nucleus and caused DNA damage and cell cycle arrest. Meanwhile, nanoribbons formed upon dephosphorylation of leucine-rich L-or D-phosphopeptide (1Lp and 1Dp) catalyzed by ALP also has nucleus targeting, the repeated stimulation of the OS cells by the peptides sensitizes the tumor cells rather than inducing resistance [233] (Fig. 9f).

The nuclear delivery of NPs requires bypassing of the cellular and nuclear membranes, but unfunctionalized NPs are mostly localized only in the cytoplasm. To further improve the targeted nuclear delivery of NPs, Bures et al. [234] combined AuNPs with cell-penetrating peptides (CPPs) and NLS. CPPs are short cationic peptide sequences that mediate the intracellular delivery of a range of biological cargo. AuNPs functionalized by CCP were allowed to be taken up efficiently into 143B OS cells, and NLS guided the way of nuclear targeting. The entry of AuNPs into the nucleus is a prerequisite for enhancing radiation therapy of primary bone tumors.

The targeting moiety strategy has promising applications. However, generic organelles such as the mitochondria and nuclei exist in all cells, both normal and tumor cells, and targeting mitochondria still has the potential to kill mitochondria within normal cells during drug delivery. Therefore, specific identification of tumor cells before organelle targeting is very necessary, which may maximize the antitumor effect.

## Passive targeting

The incomplete vascular system within the tumor tissue leads to vascular leakage with gap sizes of 100 nm to 2 μm [235, 236]. Moreover, the lack of a lymphatic system in the tumor tissues leads to poor lymphatic drainage, which results in the EPR effect [87]. The EPR effect is a prerequisite for passive targeting of nano-drug delivery systems. And drugs entering the tumor interstitium have a prolonged circulation time than normal tissues owing to higher interstitial pressure in tumor centers than in the periphery [237].

The passive targeting of nano-drug delivery systems is influenced by various factors, among which the NP particle size is crucial. Current studies have confirmed that the diameter of NPs suitable for cancer therapy should be 10–200 nm [238]. Nanoparticles with a diameter too small (< 10 nm) are easily and rapidly cleared by glomerular filtration, while they are too large to pass through the interstitial spaces between endothelial cells in the tumor vascular system [239, 240]. The polymer micelles < 100 nm are more likely to accumulate in tumor tissue. Medium-sized AuNPs (50 nm) are internalized by mammalian cells at a faster rate and a higher concentration than those of other sizes [241]. In addition, other NP properties, such as shape or surface charge, can affect the effectiveness of passive targeting. Spherical particles seem to be more endocytosed by tumor cells, whereas AuNP stars seem the most cytotoxic to human cells [242]. Electropositive NPs exhibit higher cellular uptake efficiency than negatively charged NPs [243, 244]. The inner surfaces of blood vessels and cells contain many negatively charged components that repel negatively charged NPs. As the surface charge increases (positive or negative), macrophage clearance increases, leading to greater clearance by the reticuloendothelial system [245].

In current OS research, most nano-drug delivery carriers such as liposomes, metal NPs, and magnetic NPs passively target tumor cells using the EPR effect to enhance accumulation in tumor tissue. Before active targeting was proposed, passive targeting was considered a good strategy for tumor treatment. We cannot ignore that a passive targeting strategy can achieve accumulation in tumor tissues through the EPR effect, but further tumor cell specific uptake of antitumor drugs requires active guidance of targeting ligands or receptors. Thus, we should reconsider the influence of passive targeting in future tumor targeting, which will help improve the efficiency of targeted antitumor therapy.

## Conclusions and future perspectives

This paper mainly reviews the different targeting schemes and targeting ligand selection of nano-drug delivery systems in OS therapy and describes the efficacy of targeted therapy under different ligand mediations. In the treatment of OS, nano-drug delivery systems have the unique advantage of exerting superior antitumor activity by delivering different compounds such as chemotherapy agents, photosensitizers, and immune inducers either alone or in combination. The targeting strategy ensures maximum efficiency of nano-drug delivery and bone tumor cell-killing ability. However, there is still room for refinement and improvement in those current studies.

1. Active targeting exerts targeting effects by identifying biomarkers specifically on the surface of bone tumor cells. However, most studies identify biomarkers as highly expressed markers, which are also expressed in normal cells, and the biodistribution of many nano-drug delivery vehicles still cannot reach higher enrichment in the tumor tissues. There are relatively few studies on nano-drug delivery targeting specific OS-related biomarkers; thus, there is a need to develop and design smart therapeutic agents or specific biomarkers to differentiate between normal and tumor cells to improve their cytotoxic capacity in tumor cells.
2. Current active targeting studies of OS focus on the modification of single ligands or aptamers. Unwanted off-target effects or undesired modifications of the ligand severely compromise the therapeutic efficacy of individual targeting. Therefore, multi-ligand modifications of nanocarriers could help enhance the targeting efficiency [246, 247]. Meanwhile, current ligands are only used to mediate tumor targeting of nano-drug delivery systems, and more research should explore the intracellular cascade response generated by ligand-receptor binding, or design smarter ligands such as antibodies to achieve therapeutic effects while binding to the receptor.
3. Studies have focused on primary or metastatic solid OS, whereas nano-drug delivery systems for circulating tumor cells (CTCs) have been less thoroughly investigated. As an important component of liquid biopsy, targeted capture CTCs have important diagnostic value for distant metastasis and recurrence of tumors. Current studies have explored more about how to specifically capture CTCs, and we should clarify that liquid biopsy is only a diagnostic tool, not a treatment tool. Therefore, under the premise of specific capture or identification of CTCs, nano-drug delivery systems deliver tumor-killing drugs or induce CTC apoptosis, such as CAR-T in the treatment of liquid tumors, which will significantly inhibit tumor metastasis.

In conclusion, emerging nano-drug delivery systems served as a treatment for OS offer great promise. However, research on these nanomaterials is still at the stage of cellular and animal experiments, and clinical application remains a long-term goal. We believe that nanotechnology could completely change the way cancer is detected and treated in the near future.

## Data Availability

Not applicable.
